# Genomic and Experimental Analysis of the Insecticidal Factors Secreted by the Entomopathogenic Fungus *Beauveria pseudobassiana* RGM 2184

**DOI:** 10.3390/jof8030253

**Published:** 2022-03-01

**Authors:** Fabiola Altimira, Matias Arias-Aravena, Ling Jian, Nicolas Real, Pablo Correa, Carolina González, Sebastián Godoy, Jean Franco Castro, Olga Zamora, Cristina Vergara, Nancy Vitta, Eduardo Tapia

**Affiliations:** 1 Laboratorio de Entomología y Biotecnología, Instituto de Investigaciones Agropecuarias, INIA La Platina, Santiago 8831314, Chile; matias.arias@ug.uchile.cl (M.A.-A.); nicolas.real@mayor.cl (N.R.); pcorrea1972@gmail.com (P.C.); godoygonzalez.s@gmail.com (S.G.); nvitta@inia.cl (N.V.); etapia@inia.cl (E.T.); 2 Department of Plant Protection, Institute of Vegetables and Flowers, Chinese Academy of Agricultural Sciences, Beijing 100081, China; lingjian@caas.cn; 3 Center for Bioinformatics and Genome Biology, Fundación Ciencia & Vida, Santiago 7780272, Chile; carola.mgr@gmail.com; 4 Banco de Recursos Genéticos Microbianos, Instituto de Investigaciones Agropecuarias, INIA, Chillán 3800062, Chile; jean.castro@inia.cl; 5 Laboratorio de Materias Primas y Alimentos, Instituto de Investigaciones Agropecuarias, INIA La Platina, Santiago 8831314, Chile; olga.zamora@inia.cl (O.Z.); cristina.vergara@inia.cl (C.V.)

**Keywords:** *Beauveria pseudobassiana*, entomopathogenic fungi (EPF), nonribosomal peptide synthetase (NRPS), polyketide synthase (PKS), extracellular enzymes, insecticidal activity

## Abstract

The entomopathogenic fungus *Beauveria pseudobassiana* strain RGM 2184 can reach a maximum efficacy of 80% against the quarantine pest *Lobesia botrana* in field assays. In this study, the RGM 2184 genome was sequenced, and genome mining analyses were performed to predict the factors involved in its insecticidal activity. Additionally, the metabolic profiling of the RMG 2184 culture’s supernatants was analyzed by mass spectrometry, and the insecticidal activity from one of these extracts was evaluated in *Galleria mellonella* larvae. The genome analysis resulted in 114 genes encoding for extracellular enzymes, four biosynthetic gene clusters reported as producers of insecticidal and bactericidal factors (oosporein, beauvericin, desmethylbassianin, and beauveriolide), 20 toxins, and at least 40 undescribed potential biocontrol factors (polyketides and nonribosomal peptides). Comparative genomic analysis revealed that 65–95% of these genes are *Beauveria* genus-specific. Metabolic profiling of supernatant extracts from RGM 2184 cultures exhibited secondary metabolites such as beauveriolide, oosporein, inflatin C, and bassiatin. However, a number of detected metabolites still remain undescribed. The metabolite extract caused 79% mortality *of Galleria mellonella* larvae at 28 days. The results of this research lay the groundwork for the study of new insecticidal molecules.

## 1. Introduction

Entomopathogenic fungi (EPF) are specialized microorganisms able to infect and thus reduce arthropod populations. More than 750 species of EPF, belonging to 85 genera, have been reported to date, infecting more than 1000 species of insect pests [1]. This capability allows their use as an alternative to chemical insecticides for pest control [2]. The genera *Metarhizium*, *Beauveria*, *Cordyceps*, and *Akanthomyces* are the most used for pest control because they are relatively easy to grow en masse, have a wide range of hosts, and exhibit similar efficacy to commercial insecticides [3,4,5,6].

The activity of entomopathogenic fungi is mediated by three main stages: (1) Conidia adherence and penetration, (2) colonization, and (3) extrusion to the host cadaver surface and sporulation. The first stage occurs when conidia are deposited on the epicuticle and germinate, subsequently forming an invasive appressorium, which exerts high pressure on the surface, generating mechanical rupture of the cuticle [2,7]. This fungal structure releases a cocktail of enzymes, which degrade the complex matrix of the cuticle, composed of lipids, proteins, and chitin. After cuticle penetration, EPF colonizes the hemolymph and produces a large array of biologically active secondary metabolites involved in pathogenesis and virulence [2,7]. Finally, when the insect died and the nutrients in the hemolymph are depleted, the blastospores still circulating in the hemocoel differentiate into hyphal structures. These then emerge on the outside of the insect for subsequent sporulation on the host surface [2].

Recent advances in whole-genome sequencing technologies and bioinformatics have revealed many biosynthetic gene clusters (BGCs) potentially involved in bioactive compound production (insecticides, immunosuppressors, antimicrobials, etc.). These BGCs are organized as groups of genes that encode biosynthetic enzymes implicated in the production of secondary metabolites [6]. Examples of these enzymes are nonribosomal peptide synthetases (NRPSs), polyketide synthases (PKSs), PKS–NRPS hybrids, dimethylallyl tryptophan synthases (DMATs), geranylgeranyl diphosphate synthases (GGPSs), terpene cyclases (TCs), terpene synthases (TSs), and fatty acid synthases (FASs) [6].

The number of BGCs can range from 15 to 100, depending on the fungus strain [8]. Although bioinformatic analyses allow one to predict the biosynthetic class of BGCs, it is not yet possible to predict the exact structure of the secondary metabolite synthesized or its putative biological activity [8]. To face these challenges, the secondary metabolites and exoenzymes produced by the EPF can be detected and purified from liquid cultures [9,10,11,12].

RGM 2184 is an EPF strain that has shown biocontrol activity against the quarantine pest *Lobesia botrana* during the autumn–winter season. A wettable powder formulation of *B. pseudobassiana* RGM 2184 has been shown to achieve a maximum efficacy level of 80% against the pupae of *L. botrana* in field trials performed in two regions of Chile over two seasons [3]. In order to obtain an approximation of the molecular basis of its entomopathogenic activity, the objectives of this study were: (1) To identify possible genes and/or gene clusters in the genome of *Beauveria pseudobassiana* RGM 2184 involved in its biocontrol activity; (2) to evaluate orthologs of these genes in other EPFs through comparative genomic analysis; and (3) to identify and evaluate insecticidal activity of secondary metabolites extract secreted by the strain RGM 2184.

## 2. Materials and Methods

### 2.1. Genome Sequencing and Assembly

The strain *B. pseudobassiana* RGM 2184 was provided by the Chilean Collection of Microbial Genetic Resources (CChRGM, Chillán, Chile) and grown on potato dextrose agar plates for 7 days at 25 °C. Then, 20 mL of spores and mycelia from this culture were harvested and subjected to DNA purification using the Wizard^®^ Genomic DNA Purification Kit (Promega Corporation, Madison, WI, USA) according to the manufacturer’s protocol. The DNA concentration was measured by a Qubit 2.0 fluorometer (Life Technologies, Carlsbad, CA, USA). The DNA integrity and purity was measured by the 5400 Fragment Analyzer System (Agilent, Santa Clara, CA, USA). The genome of the strain RGM 2184 was sequenced by Novogene (Davis, CA, USA) using the Nanopore and Illumina platforms. Oxford Nanopore sequencing was performed on PromethION. A total of 141,949 reads were achieved for this sequencing, yielding 1,357,479,554 bases and an average read length of 9429 bases. The Illumina sequencing libraries were prepared according to the Nextera XT DNA sample preparation protocol (Illumina, San Diego, CA, USA). The DNA was sequenced using an Illumina HiScanSQ sequencer, according to the paired-end approach. The Illumina sequencing resulted in 8,584,850 paired-end reads with a length of 250 bp. The genome was assembled using Canu version 1.6 [13] and this initial assembly was polished based on nanopore reads using racon version 1.4.3 with the default parameters [14].

### 2.2. Genome Annotation

De novo prediction of gene functionalities was conducted using three different suites of software: Genemaker [15], GlimmerHMM V3.0.4 [16], and SNAP [17]. Augustus V2.7 [18] was employed for gene prediction based on homologous genes, using model training based on coding sequences downloaded from NCBI. Functional annotation was carried out using the Swissport, Pfam, KEGG, GO, COG(KOG), and CAZyme databases. Potential secreted proteins were predicted by WoLF PSORT [19,20] and SignaIP 3.0 [21,22]. To identify the gene clusters encoding PKS and NRPS, responsible for the biosynthesis of secondary metabolites, the whole genome data set was subject to antiSMASH analysis [23,24]. Circular genome visualization of RGM 2184 was achieved using the DNAPlotter application of Artemis software version 18.0.2 [25] and edited using Inkscape version 1.1.1.

### 2.3. Phylogenetic Analysis

Multiprotein species trees were built from 25 concatenated ribosomal protein families [26] from 25 entomopathogenic strains, which were associated with a COG category (Table S1). Ribosomal proteins were recovered through assignation of predicted protein-coding genes to COG classification by the genomic tool eggNOG-mapper [27,28]. The alignment of concatenated sequences was made using MAFFT with L-INS-I iterative refinement [29], which were masked to remove unreliable aligned regions using GBLOCKS [30,31]. A maximum likelihood tree was prepared for concatenated alignments with IQ-TREE [32] using 1000 replicates as bootstrap and the best substitution model proposed by the same tool.

### 2.4. Comparative Genomic Analysis

The Average nucleotide identity (ANI) and digital DNA–DNA hybridization (dDDH) were used to evaluate the taxonomic relatedness between *B. pseudobassiana* RGM 2184 and 27 EPF strains sequenced (Table S2). ANI is defined as a pairwise measure of overall similarity between two genome sequences [33]. Identity values were calculated between fragments of EPF (query) and homologous regions of the *B. pseudobassiana* RGM 2184 genome (subject). The final ANI value is the mean of identity values of all fragments of the query genome. The ANIb (ANI algorithm using BLASTN) algorithm described by Goris et al. 2007 [34] was used in the analysis of this study. This algorithm is available at the following link: http://jspecies.ribohost.com/jspeciesws/#analyse (accessed on 8 November 2021) [35]. Also, dDDH was per-formed between sequenced entomopathogenic fungi (query) and *B. pseudobassiana* RGM 2184 genome (subject) using Genome-to-Genome Distance Calculator (GGDC) 3.0 software available at the following link: https://ggdc.dsmz.de/ggdc.php (accessed on 3 January 2022) [36]. GGDC software is underlying principle is as follows: two genomes A and B are locally aligned using tools such as BLAST [37], which produce a set of high-scoring segment pairs (HSPs; these are intergenomic matches). In the second step, information contained in these HSPs (e.g., the total number of identical base pairs) is transformed into a single genome-to-genome distance value using a specific distance formula [37].

A BLAST score ratio (BSR) test [38] was conducted to compare the homologous gene-encoding exoenzymes PKS and NRPS between *B. pseudobassiana* RGM 2184 and the sequenced entomopathogenic fungi. Each gene-encoding exoenzymes PKS and NRPS of *B. pseudobassiana* RGM 2184 was aligned against each sequenced entomopathogenic fungi (query) including RGM 2184 (reference) strain with BLASTN and the query bit score was tabulated. The BSR value was calculated by dividing the query bit score by the reference bit score, resulting in a BSR value between 0.0 and 1.0 [38]. A score of 1 indicates a perfect match, while a score of 0 indicates no BLAST match of a query gene in the *B. pseudobassiana* RGM 2184 genome. Values over 0.4 indicate the presence of a gene homologue [38]. The normalized pairs of BSR indices were plotted using R software version 4.0).

### 2.5. Regression and Correlation Analysis

Linear regression was carried out using 27 values of ANIb (%) or digital DDH (dDDH) (%) previously obtained by the comparison between RGM 2184 and 27 EPF strains (Section 2.4). These values were used as dependent variables and the degree of taxonomic relatedness between RGM 2184 and the 27 EPF strains as the independent variable. The values 1, 2, 3, 4, and 5 indicate that the highest degree of taxonomic relatedness between RGM 2184 and the EPF strains is at the species, genera, family, order, and subfamily levels, respectively. For interpretation of the analysis, an R-squared value close to 100% indicates a good fit of the data to the model, giving a high Pearson product-moment correlation (close to 1) between the dependent and independent variables. Pearson product-moment correlations between variables ANIb (%), dDDH (%), toxins, exoenzymes, NRPS, and PKS were calculated. Correlation coefficients close to 1 indicate a highly positive linear relationship, while those close to −1 indicate a highly negative linear relationship, and those close to 0 indicate no linear relationship. The analyses were carried out using Statgraphics Centurion XVII version 17.1.02.

### 2.6. Extraction of Secondary Metabolites from the Supernatant of RGM 2184 Cultures

Conidia from PDA cultures at seven days old were suspended in sterile 0.9% NaCl supplemented with 0.05% Tween 80 (Sigma-Aldrich, St. Louis, MO, USA). A 10^6^ conidia/mL suspension was used to inoculated in 250 mL Erlenmeyer flasks containing 83 mL of an M2 [39] and YSM [40] liquid medium, respectively. The cultures were incubated at 150 rpm and 25 ± 2 °C for three days. These cultures were used to inoculate a 7 L stirred tank reactor (ez-Control, Applikon Biotechnology, Delft, Netherlands) containing 2 L of M2 and YSM medium, respectively. The aerated cultures were stirred (200 to 800 rpm) for three days at 25 °C. Subsequently, the cultures were centrifuged to 10,000× *g* for 10 min. The supernatants were filtered using a 0.45 µm filter unit (Millipore, Burlington, MA, USA), and the filtered supernatants were adjusted to pH 2.0 with 37% (*w*/*v*) HCl [41]. Secondary metabolites were extracted two times with an equal volume of ethyl acetate. The extracts were dried using the rotary evaporator Bath B-100 (Büchi, Flawil, Switzerland) under a reduced pressure evaporator. The dried extracts were resuspended in methanol. Finally, the resuspension solvent of the extracts was completely evaporated using a SpeedVac vacuum concentrator (Savant SPD 121P, Thermo Scientific, Waltham, MA, USA).

### 2.7. Detection and Identification of Compounds by Mass Spectrometry

The extracts obtained from the supernatant of the M2 and YSM media were analyzed by HPLC 1200 Rapid Resolution chromatograph (Agilent Technologies Inc., Santa Clara, CA, USA) coupled to a mass spectrometer with an electrospray ionization source and a maXis time-of-flight mass analyzer (MS-ESI-TOF Bruker Daltonik GmbH, Bremen, Germany). A Zorbax SB-C8 column (2.1 × 30 mm, 3.5 µm particle size) was used for the chromatography. Extract fractions were eluted using a solvent gradient (solvent A, water/acetonitrile 90:10 and solvent B, water/acetonitrile 10:90, both with a 13 mM concentration of ammonium formate and 0.11% trifluoracetic acid). The flow rate was kept at 3 mL/min at room temperature. The mass spectrometer was set to positive ESI mode. For samples from the M2 medium, only information for intact molecules (MS spectra) was acquired, while for sample from YSM medium, intact molecules and their fragmentation patterns (MS and MS/MS spectra) were acquired. DataAnalysis version 4.4 (Bruker Daltonik GmbH) was used to visualize the chromatograms and mass spectra. The *m*/*z* signals were selected according to their intensity (discarding all precursor *m*/*z* signals with an intensity lower than 100). Subsequently, an identification analysis was performed by comparing the selected *m*/*z* signals to the one available in the AntiBase database: The Natural Compound Identifier 2012 (Wiley-VCH, Weinheim, Germany) [42]. The Sirius tool version 4.8.2 was used for the MS/MS analysis of metabolites from the supernatant of the RGM 2184 strain culture in YSM medium [43].

### 2.8. Secondary Metabolite Extract Effect on Galleria Mellonella Mortality

The powdered extract from the supernatant of the RGM 2184 strain culture in YSM medium was resuspended in 50 mM Tris-HCl buffer (pH 6.0) to a concentration of 1 mg/mL. Serial dilutions of this concentration were made in a range of 0.1–1.0 mg/mL (0.1, 0.25, 0.5, 0.75, and 1 mg/mL). Aliquots of 7 µL of each extract dilution were directly injected into the abdominal region of *G. mellonella* larvae through a last pro-leg (7–0.7 µg/larva) [44]. The buffer, 50 mM tris–HCl (pH 6.0), was injected as a control. Ten larvae were injected for each treatment. Post-injection larvae were incubated in Petri dishes at 25 ± 1 °C. Each treatment was conducted with three replicates, and the experiment was independently repeated four times. Mortality, color change, and state of development were recorded every seven days for a month. The data obtained from the four independents experiments were subjected to an ANOVA using a *p*-value of <0.05 as the cutoff. The means were separated using an LSD test at the 5% significance level using Statgraphics Centurion XVII version 17.1.02.

## 3. Results

### 3.1. Genomic Features of the RGM 2184 Strain

The genome of *B. pseudobassiana* RGM 2184 was shotgun sequenced to 60× coverage using the Oxford Nanopore and Illumina platforms. The parameters of coverage and scaffold N50 of the RGM 2184 genome assembly were better than most of the entomopathogenic fungi sequenced (Table S2). The predicted genome of RGM 2184 has the same sizes as *B. pseudobassiana* KACC 47,484 (34.5 Mb) and similar to the other EPF strains sequenced (27.6–43.3 Mb), showing a G + C content percentage of 51.7% and encoding 8469 proteins. The number of protein-encoding genes was lower than almost all of the EPF strains (Table S2).

### 3.2. Identification of the RGM 2184 Strain

**Figure 1:**
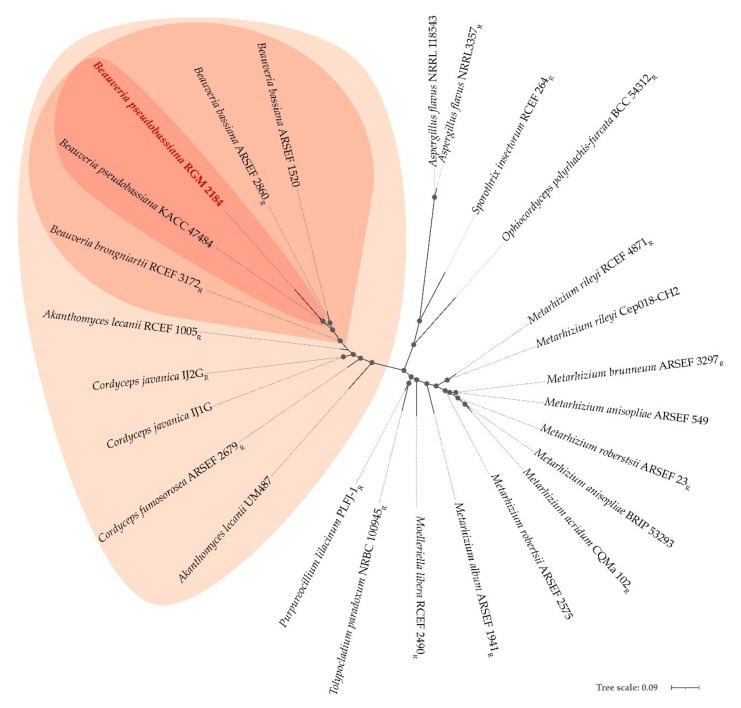
Maximum-likelihood phylogenomic tree of the EPF strains based on universally shared concatenated sequences. The tree was constructed with 25 conserved sequences using IQ-Tree, with bootstrap percentages ≥60% shown at nodes. The bar represents 0.09 amino acid substitutions per site. The RGM 2184 strain is written in red. The strains that belong to same species, genera, and family as RGM 2184 are enclosed in dark red, light red, and lightest red, respectively. R, representative genome published in NCBI.

**Figure 2:**
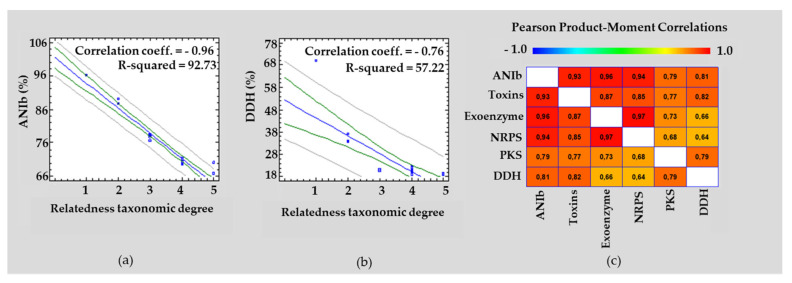
Correlation analysis of the parameters evaluated in the RGM 2184 and 27 EPF strains. Regression analysis of ANIb (%) (**a**) and dDDH (%) (**b**) versus the different degrees of taxonomic relatedness between the RGM 2184 and 27 EPF strains. The values 1, 2, 3, 4, and 5 indicate that the highest degrees of taxonomic relatedness between the RGM 2184 and EPF strains are at the species, genera, family, order, and subfamily levels, respectively. (**c**) of Person product-moment correlation coefficient graph between the variables ANIb (%), dDDH (%), homologous gene encoding toxins, exoenzymes, PKS, and NRPS. Values of the correlation coefficient close to 1 indicate a highly positive linear relationship (red color). Blue line indicates trendline of data set. Green lines indicate confidence intervals for the mean response at X. Gray lines indicate prediction limits for new observations.

A phylogenetic tree for the RGM 2184 strain and the 27 other *EPF strains* was built using a concatenated alignment of 28 universal ribosomal proteins. This analysis indicated that the RGM 2184 strain belongs to the *B. pseudobassiana* clade (Figure 1). Additionally, dDDH and ANIb analyses showed values of 96% and 70%, respectively, between the RGM 2184 and *B. pseudobassiana* KACC 47,484 strains (Table 1). The proposed and generally accepted species boundary for ANI and dDDH values are 95~96% and 70%, respectively [45]. Therefore, these parameters support the belonging of RGM 2184 to the *B. pseudobassiana* species. It should be noted that the dDDH values obtained for the EPF strains belong to the same genus, but different species drastically decreased to 34–37%. Meanwhile, the values of ANIb decreased to 88–89% (Table 1). The correlations between the percentages of ANIb and dDDH of RGM 2184 regarding the 27 EPF strains belonging to different degrees of taxonomic relatedness (species (1), genera (2), family (3), order (4), and subphylium (5)) were −0.96 and −0.76, respectively (Figure 2a,b).

### 3.3. Extracellular Enzymes Encoded in the Genome of B. peudobassiana RGM 2184

**Figure 3:**
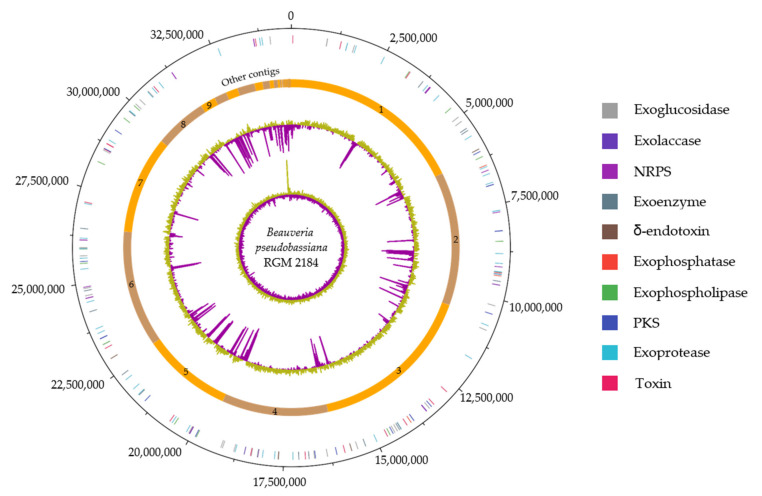
Circular representation of *Beauveria pseudobassiana* RGM 2184. Tracks from outside to inside: Circle 1, nucleotide base position (bp) clockwise, starting from zero; circle 2, selected protein-encoding regions; circle 3, biosynthesis gene clusters detected are indicated by the lime-colored regions; circle 4, position of DNA contigs, light orange = odd-numbered contig, dark orange = even-numbered contigs; circle 5, G + C nucleotide content plot, using a 10 kb window size, with lime/purple peaks indicating values higher/lower than the average G + C content, respectively; circle 6, GC skew plot [(G − C)/(G + C)], using a 10 kb window size, with lime/purple peaks indicating values higher/lower than 1, respectively.

**Figure 4:**
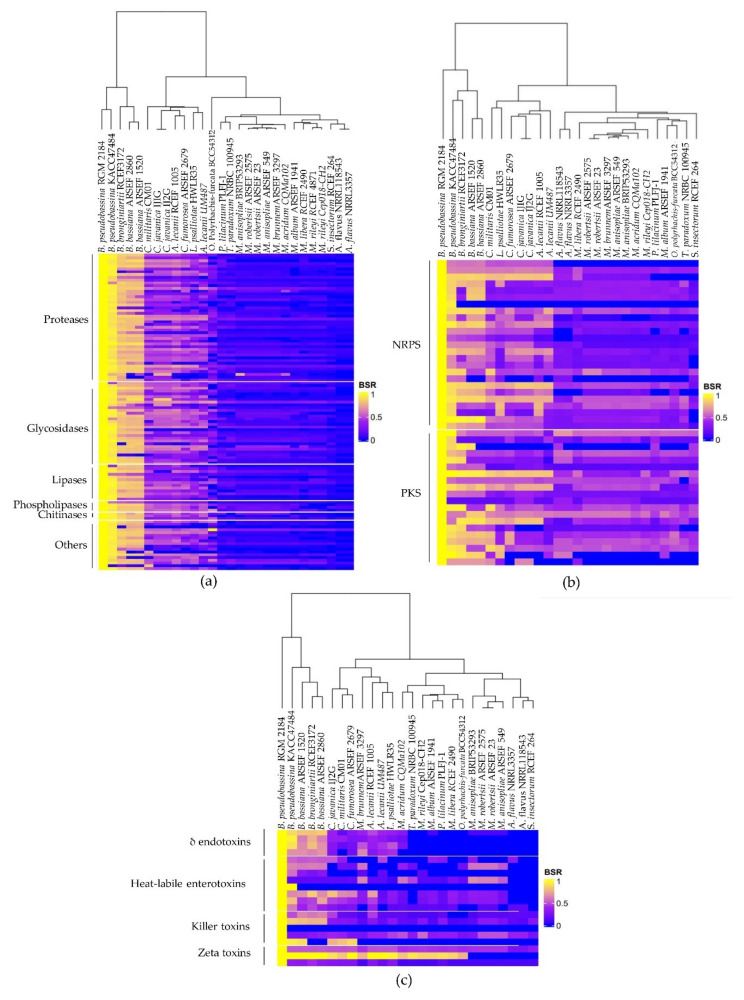
Heat maps displaying the homology levels between PKS, NRP, and the exoenzyme- and endotoxin-encoding genes of *B. pseudobassiana* RGM 2184 in comparison to other entomopathogenic fungi. A score of 1 indicates a perfect match, while a score of 0 indicates no BLAST match of a query gene in the reference genome. Values over 0.4 indicate the presence of a homologous gene. (**a**) Comparative analyses of extracellular enzymes. (**b**) Comparative analyses of NRPS and PKS. (**c**) Comparative analyses of toxins.

The genome of the RGM 2184 strain encodes 114 extracellular enzymes (Table S3). These enzymes are mainly proteases (40%), glycosidases (26%), lipases (11%), chitinases (3%), phospholipases (3%), and laccases (2%), which could be involved in the colonization of insects. These genes are distributed throughout the chromosome (Figure 3). Of them, 98%, 93–95%, and 38–65% show homology with genes of strains that present the highest degree of taxonomic relatedness regarding to RGM 2184 at the species, genera, and family levels, respectively (Table 1 and Figure 4a). These results are consistent with the genomic comparative analysis between the RGM 2184 and EPF strains, showing a high correlation (0.96) between the percentages of ANIb and the homologous genes encoding the exoenzymes in theses strains (Figure 2c).

### 3.4. NRPS and PKS Encoded in the Genome of B. peudobassiana RGM 2184

The bioinformatic prediction of BGCs indicated that RGM 2184 encodes 25 NRPSs and 20 PKSs (Figure 3 and Table S4). A comparative genomic analysis between the RGM 2184 and 27 EPF strains indicated that there is a correlation of 0.94 and 0.74 between the percentages of ANIb and the homologous genes encoding NRPSs and PKSs, respectively (Figure 2c). Of the NRPS-encoding genes, 100%, 80–88%, and 56–64% showed homology with the genes of the EPF strains presenting the highest degree of taxonomic relatedness regarding the RGM 2184 strain at the species, genera, and family levels, respectively (Table 1 and Figure 4b). Meanwhile, for the PKS-encoding genes, 95%, 80–90%, and 40–70% of them showed homology with the genes of those strains presenting the highest degree of taxonomic relatedness regarding the RGM 2184 strain at the species, genera, and family levels, respectively (Table 1 and Figure 4c).

**Figure 5:**
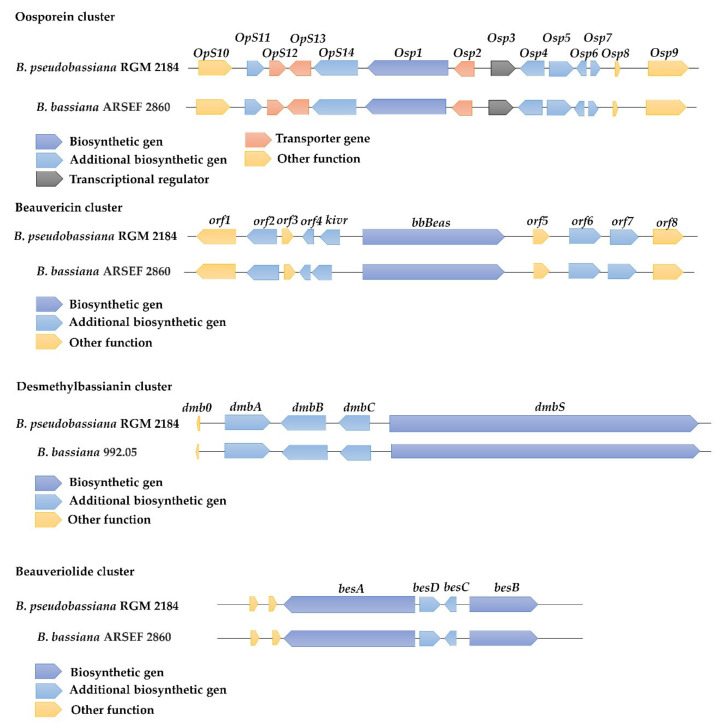
Gene clusters of the RGM 2184 strain involved in bioactive compound biosynthesis. Comparisons of PKS (oosporein), NRPS (beauvericin and desmethylbassianin), and NRPS-PKS (beauveriolide) clusters in RGM 2184 (**top**) and *B. bassiana* ARSF 2860 or *B. bassiana* 992.05 (**bottom**).

Four putative biosynthesis clusters, highly conserved in *Beauverias* spp., were found in the genome of the RGM 2184 strain. These clusters participate in the synthesis of oosporein, beauvericin, desmethylbassianin, and beauveriolide (Figure 5 and Table S5). The oosporein biosynthetic cluster (37 Kb) comprises at least seven genes, including a non-reducing polyketide synthase (oosporein synthase 1 (OpS1)), a membrane transporter (OpS2), a transcription factor (positive regulator, OpS3), and four additional enzymes involved in oosporein biosynthesis (OpS4–OpS7). The beauvericin gene cluster (19 kb) encodes an NRPS (BbBEAS) and NADPH-dependent 2-ketoisovalerate reductase (KIVR). The last one converts 2-ketoisovalerate from valine catabolism or pyruvate metabolism into D-Hiv. This substrate and L-phenylalanine (L-Phe) are converted into a cyclooligomer depsipeptide called beauvericin by BbBEAS. The desmethylbassianin cluster (34 kb) includes four genes, named *dmbS*, *dmbC*, *dmbA*, and *dmbB*. The *dmbS* gene encodes desmethylbassianin synthetase, a polyketide synthase that is fused to a single module of a nonribosomal peptide synthetase (PKS-NRPS), while the *dmbC*, *dmbA*, and *dmbB* genes participate in the synthesis of desmethylbassianin from predesmethylbassianin A. Finally, the *besABCD* cluster (17 kb) is composed of four genes, *besA* (NRPS), *besD* (acyl-CoaA ligase), *besC* (acyltransferase), and *besB* (PKS). These genes participate in the synthesis of beauveriolides.

### 3.5. Bacterial- and Yeast-Like Toxins Encoded in the Genome of B. peudobassiana RGM 2184

The genome of RGM 2184 encodes 15 bacterial-like toxins that could be involved in its entomopathogenic activity (Figure 3 and Table S6). Four of them show a δ-endotoxins domain in their N-terminal described in the Cry proteins of *Bacillus thuringiensis*. Eight of them contain heat-labile enterotoxin domain, while three encode the Z-toxin domains described in enterobacteria toxins. Additionally, the genome of RGM 2184 encodes five proteins that exhibit a chitinase catalytic domain (GH18) and chitin-binding modules (CBMs) described in the killer toxin zymocin produced by the yeast *Kluyveromyces lactis*. Comparative genomic analysis indicated that there is a high correlation between the percentages of ANIb–DDH and the homologous genes encoding toxins (Figure 2c). Of the toxin-encoding genes, 80%, 65–70%, and 25–30% show homology with the genes of the strains that presented the highest degree of taxonomic relatedness regarding RGM 2184 at the species, genera, and family levels, respectively (Figure 4c and Table 1).

### 3.6. Detection and Identification of Secondary Metabolites in the Supernatant of the Cultures

The RGM 2184 strain was grown in two culture media (M2 and YSM). The color of both culture media changed considerably over time (Figure S1a). Analysis of the supernatant extracts of the cultures indicated that the RGM 2184 strain released a significant number of compounds (Tables S7–S9). The molecular mass and retention time corresponding to each *m*/*z* are indicated in Tables S7–S9. In the MS spectra of both culture media, a similar profile was observed in the 0–5 min separation interval, while a greater difference was shown in the 5–10 min interval (Figure S1b,c). Identification analysis of the supernatant of the M2 culture extract based on *m*/*z* signals indicated the presence of dipicolinic acid (C_7_H_5_NO_4_), inflatin C (C_9_H_14_O_7_), beauveriolide V/VI (C_23_H_41_N_3_O_5_), beauverolide H/I/III (C_22_H_41_N_5_O_7_), mycosporine–alanine (C_11_H_17_NO_6_), catathelasmol E (C_9_H_16_O_5_), and 14 other compounds (Table S7). Meanwhile, in the YSM medium, oosporein (C_14_H_10_O_8_), orsellinic acid (C_8_H_8_O_4_), bassiatin (C_15_H_11_NO_6_), an intermediary of dipicolinic acid (4-methyl-2,6-pyridinedicarboxylic acid (C_8_H_7_NO_4_), dimethyl-2,6-pyridinedicarboxylic acid (C_9_H_9_NO_4_), and dimethyl 4-(3-methoxy-2-methoxycarbonyl-3-oxopropyl)pyridine-2,6-dicarboxylic acid (C_15_H_17_NO_8_)) and mycosporine-alanine (C_11_H_17_NO_6_) were detected (Tables S8 and S9). Another 20 and 33 peaks were detected in the MS and MS/MS analyses, respectively, from an extract of RGM 2184 cultures in YSM medium, but their identification could not be determined by comparison of *m*/*z* signals from the databases.

### 3.7. Insecticidal Activity of the Metabolite Extract against G. mellonella Larvae

**Figure 6:**
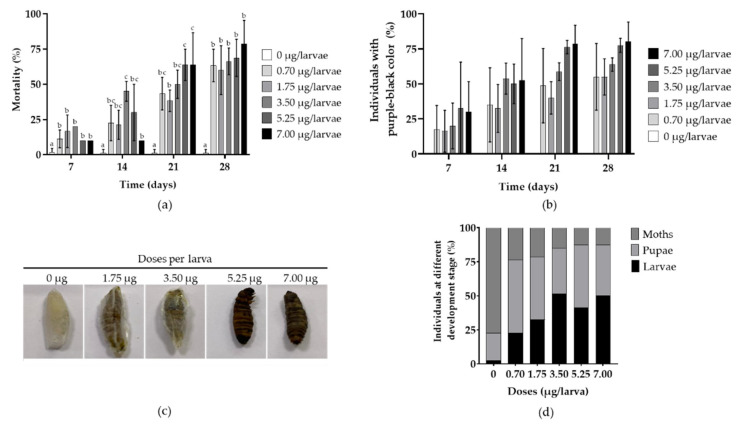
Evaluation of mortality, color change, and developmental stage of *G. mellonella* larvae in the treatments inoculated with different doses of the metabolite extract. (**a**) Mortality. (**b**) Color change. (**c**) Effect of the metabolite extract from the supernatant of the *B. pseudobassiana* RGM 2184 cell culture on *G. mellonella* larvae after 21 days post-injection. (**d**) Percentage of different developmental stages of individuals in the treatments (moth, pupa, and larva). Ten fifth instar larvae of *G. mellonella* were injected with different doses of the secondary metabolite extract. The control treatment (0 μg/larva). The averages of the values ± SD of the treatments performed in triplicates are expressed. Different letters indicate significant differences (*p* < 0.05, ANOVA, LSD).

*G. mellonella* larvae were injected with five different doses (0.70, 1.75, 3.50, 5.25, and 7.00 µg) per larva of extract enriched with metabolites secreted by RGM 2184 (Figure 6). After seven days, the experimental treatments exerted 10–20% larva mortality (Figure 6a). Additionally, between 16% and 30% of the individuals showed a color change from yellow-brown to purple-black (Figure 6b). Meanwhile, in the control treatment, 96% of individuals remained alive, and without color change. After 21 days of incubation, there was a significant increase in larva mortality (Figure 6a) and in the number of individuals with color change (Figure 6b,c). The treatments of 7.00, 5.25, 3.50, 1.75, 0.70, and 0.00 µg/larvae showed a mortality of 64% (±23%) 64% (±11%), 50% (±10%), 38% (±8%), 43% (±11%), and 1% (±2%), respectively (Figure 6a). Additionally, 79% (±13%), 76% (±5%), 59% (±6%), 40% (±11%), 49% (±27%), and 0% of individuals showed color changes and dehydration, respectively (Figure 6b). At 28 days, the experimental treatments showed between 60% (±17%) and 79% (±16%) mortality, while the control showed only 1% (±2%) mortality (Figure 6b). Moreover, 75% of the control individuals reached the moth stage, while in the experimental treatments, only 13–24% of the individuals became moths (Figure 6d).

## 4. Discussion

The identification of *B. peudobassiana* RGM 2184 was previously performed by Altimira et al. (2022) [3] using multilocus analysis of only four molecular markers (bloc, tef, rpb1, and rpb2). In this study, taxonomic identification of this strains was confirmed using a phylogenetic analysis of 25 universal ribosomal proteins (Figure 1), together with nucleotide similarity parameters such as ANI and dDDH (Table 1). These latter parameters are widely used in the prokaryotic isolate identification, but are poorly used in the identification of eucaryotic microorganism isolates [46,47]. Usually, genomes of prokaryotic isolates belonging to the same species possess dDDH and ANIb values ≥70% and ≥95–96%, respectively [45]. These cutoff values are consistent with the phylogenetic analysis, allowing the identification of RGM 2184. Therefore, the cutoff value in prokaryotic analysis could be extrapolated to fungi. Moreover, there were high correlations between the percentage of ANIb and the degree of taxonomic relatedness between RGM 2184 and the 27 EPF strains analyzed herein (Figure 2a). This correlation was better than the percentage of dDDH regarding the degree of taxonomic relatedness (Figure 2b). These results suggest that the percentages of ANIb and dDDH can be widely used in fungus identification, and ANIb could be used in extensive taxonomic analyses. The use of this parameter could foster an increase in the number of sequenced fungi genomes, especially of ex-type fungi strains. In this study, a comparative genomic analysis of the genes encoding for the insecticidal factors found in the RGM 2184 strain with those reported in the genomes of 27 EPF strains was performed. The factors corresponded to the extracellular enzymes, NRPS, PKS, and bacterial- and yeast-like toxins involved in insect colonization. The first barrier that entomopathogenic fungi face when colonizing insects is the cuticle. Insect cuticles are a layered, fibrous composite of chitin, water, protein, catechol, lipids, and occasionally metals and minerals, secreted by a single layer of epidermal cells that serves as a morphological active defense mechanism against predatory attacks [48]. The EPF must produce and secrete hydrolytic enzymes to successfully penetrate through the cuticle [49]. The predictive genomic analysis suggested that *B. pseudobassina* RGM 2184 can release a wide variety of enzymes to degrade the cuticles and cocoons of insects (Figure 4a and Table S3). This enzymatic battery is composed mainly of proteases, glycosidases, and lipases. To date, no extracellular enzymes have been characterized in *Beauveria pseudobassiana*. However, some of them have been described in a phylogenetically close species, namely, *Beauveria bassiana*. The findings regarding extracellular enzymes described in *B. bassiana* strains provide a good approximation for *B. pseudobassiana* RGM 2184 due to the genomic comparative analysis indicating that there is a significant percentage of homologous genes encoding extracellular enzymes in strains belonging to the same genus and species (Figure 3 and Table 1). The number of homologous extracellular enzymes was significantly reduced when the highest degree of taxonomic relatedness between RGM 2184 and EPF strains was at the family or order level (Figure 4a and Table 1).

Once the fungal hypha penetrates the insect cuticle and invades the hemocoel, it multiplies as a blastospore. This type of cellular structure has a thin cell wall and presents a yeast-like form, offering a great surface/volume ratio for absorption of nutrients from the hemolymph (insect blood) [2]. The EPF blastospore also secretes abundant organic compounds of lower molecular mass called secondary metabolites. These are necessary for both suppressing an insect’s defense systems and impeding opportunistic microorganism propagation [50]. In the genome of RGM 2184, we found clusters of known genes involved in the synthesis of oosporein, beauvericin, desmethylbassianin, and beauverolides (Figure 5). Oosporine is a red dibenzoquinone pigment initially reported in *Oospora colorans* and subsequently found in several soil and endophytic fungi, as well as in the entomopathogenic *Beauveria* genus [41]. Fan et al. (2017) demonstrated that oosporein production is induced during the late stages of infection, but not in the early stages, including attachment, penetration, proliferation, and immune evasion during hyphal body growth in the host hemocoel. Oosporein has been shown to display strong antimicrobial activity toward host bacterial flora, inhibiting the ability of bacteria to proliferate on cadavers [41]. Beauvericin is a cyclic hexadepsipeptide that belongs to the enniatin antibiotic family. It has been described in many fungi, such as *Beaveria bassiana* and *Fusarium* spp. Beauvericin has strong insecticidal activity against a broad spectrum of insect pests, such as *Calliphora erythrocephala*, *Aedes aegypti*, *Lygus* spp., *Spodoptera frugiperda*, and *Schizaphis graminum* [51]. Additionally, beauvericin has strong antibacterial activity against human, animal, and plant pathogenic bacteria [51]. Desmethylbassianin (Dmb) has been described in some strains of *Beauveria bassiana*. The structure of Dmb differs from bassianin by the lack of a methyl group. It has been shown that bassianin inhibits membrane ATPase activity in erythrocytes, causing varying degrees of cell lysis [52]. However, Dmb’s mechanism of action is not yet clear.

In contrast to entomopathogenic bacteria and viruses, entomopathogenic fungi infect insects via cuticular penetration and are usually assumed to lack oral infectivity. However, the RGM 2184 strain encodes bacterial-like toxins such as enterotoxin and γ-toxin, suggesting a potential oral toxicity (Table S6). Similar findings have been found through genomic and transcriptomic analysis of other entomopathogenic fungi strains. For example, *B. bassiana* ARSEF 2860 genome encodes seven Cry protein-like toxins, 13 heat-labile enterotoxins, and three zeta toxins [53]. On other hand, *B. bassiana* Bb8028 strain possess five genes encoding bacterial-like toxins. These genes were highly expressed in fungal infection of this strains in mosquitoes [54]. Ortiz-Urquiza (2021) [55] proposes that these genes alter insect gut epithelial permeability, facilitating noncanon-ical routes (cuticle penetration route) of infection via the gut after the ingestion of fun-gal spores. Alternatively, zeta toxins could act as bactericidal agents during fungal necrotrophic/saprophytic growth. Additionally, the genome of RGM 2184 encodes killer toxin-like chitinases, similar to that described in the yeast *Kluyveromyces lactis*. This protein could have fungal antagonism by permeabilization of fungal cell walls to allow penetration of antifungal molecules [56].

Comparative genomic analysis between EPF strains indicated that there is a significant percentage of homologous genes encoding NRPS, PKS, and toxins in strains that belong the same genus and species as RGM 2184 (Figure 4 and Table 1). The number of homologous NRPSs, PKSs, and toxins was significantly reduced when the highest degree of taxonomic relatedness between RGM 2184 and the EPF strains was at the family or order level (Figure 4 and Table 1).

These results suggest that there is a wide diversity of NRPSs and PKSs involved in the synthesis of secondary metabolites in the family’s taxonomy belonging to the order Hypocreales, such as *Cordycipitaceae* (e.g., *Beauveria* ssp.) and *Clavicipitaceae* (e.g., *Metarhizum* spp.). This background is relevant in a biocontrol strategy because a mixture of strains belonging to *Beauveria* and *Metarhizium* genera could have a different enzymatic and metabolite repertoire to exert their action on the pest. This could contribute to a reduced possibility of pest resistance.

It has been demonstrated that several entomopathogenic fungi secrete insecticidal, anti-feedant, or toxic bioactive compounds in liquid cultures, which can be purified [9,11]. For example, Spinosad and Abamectin are two widely used commercial insecticides based on the culture harvesting and purification of microbial metabolites [10]. The RGM 2184 strain secretes a wealth of secondary metabolites into the culture medium. Of these, inflatin C, orsellinic acid, oosporein, bassiatin, picolinic acid, beauveriolide V/VI, and beauveriolide H/III/I were identified and described in EPF fungi (Tables S7–S9). Inflatin C has been described in the ascomycete fungus cyclosporin-producing strain of *Tolypocladium inflatum* (ATCC 34921), a pathogen of beetle larvae. Studies of the cytotoxicity of inflatin C against nine human tumor cell lines have shown no cytotoxic effect at a concentration of 100 µM [57]. No other antecedents have been reported in the literature for the action of inflatin C. Orsellinic acid is an intermediate of oosporein, and both compounds were predicted through informatic analysis of the genome of RGM 2184. Bassiatin has been isolated from *Beauveria bassiana* K-717 [58] and *Fusarium oxysporum* J8-1-2 culture medium [56]. This compound inhibited ADP-induced aggregation of rabbit platelets with an IC_50_ of 1.9 × 10^–4^ M. Other research has found that bassiatin inhibits cell proliferation and cell cycle progression by repressing cyclin D1 [59]. In contrast, beauverolide was initially isolated and characterized from the entomopathogenic fungus *Beauveria tenella* in 1975 [58]. Currently, there are 28 structurally different beauveriolides that have been identified. Congeners of beauveriolides have been detected in insect hemolymphs after *B. bassiana* infection. Beauveriolide I has moderate insecticidal activity [60], while injection of wax moth (*Galleria mellonella*) larvae with beauverolide L does not kill insects at a dosage of less than 30 µg per larvae, but does induce immune responses in insects [61]. It is still unclear whether beauveriolide production is required for full fungal virulence against insect hosts. Additionally, different medicinal activities have been reported for beauveriolides [62], including antiaging [63], beta-amyloid lowering, and inhibition of acyl coenzyme A (acyl-CoA)/cholesterol acyltransferase activity to block the synthesis of cholesteryl esters [64,65].

*G. mellonella* has previously been used as a model system to study the toxicity of Bsp70 toxin [10] and to evaluate the effect on the suppression of the compound Cyclosporin A on the humoral immune response of insects [44]. In our study, we used fifth instar larvae of *G. mellonella* to study the toxicity effect of the metabolite extract secreted by RGM 2184. The results of larvae injection with the metabolite extract showed no dose-dependent toxicity effect, and larva mortality in the experimental treatments increased significantly after 14 days post-injection (Figure 6a). Larvae inoculated with the extract started to turn purple/black after 7–21 days post-injection (Figure 6b). This color change is characteristic of the melanization process, a known insect immune response resulting from the conversion of the inactive form of pro-phenol oxidase enzymes into active phenol oxidase to limit the distribution of a foreign agent within the insect hemocoel [10]. Additionally, a low percentage of larvae from the experimental treatments completed their developmental cycle compared to the control. These results could be because the extract possesses a heterogeneous mixture of more than 20 compounds that may have different properties (insecticidal, antimicrobial, immunosuppressive, growth modulating, etc.) that ultimately led to a significant increase in mortality, along with a heightened response of the immune system (melanization) and a cessation of the developmental cycle (Figure 6d).

## 5. Conclusions and Outlook

The results of this study suggest that percentages of ANIb and dDDH could be widely used in fungi identification at the species level and, additionally, ANIb could be used extensively for taxonomic analysis. The genome of *B. pseudobassiana* RGM 2184 exhibits a broad range of genes encoding extracellular enzymes, biosynthetic gene clusters, and bacterial- and yeast-like toxins that could be involved in its insecticide activity. Most of these genes have been poorly characterized or have not been previously described.

The genomic comparative analysis herein indicated there is a significant percentage of genes encoding homologous exoenzymes, BGCs, and bacterial- and yeast-like toxin genes in strains that belong to the same genus and species. There is a wide diversity exoenzymes and BGCs involved in the synthesis of secondary metabolites associated with the genomic diversity of the order Hypocreales. This is relevant in a biocontrol strategy, because a mixture of strains belonging to *Beauveria* and *Metarhizium* would have a different insecticidal factors repertoire. This could contribute to a reduced possibility of pest resistance.

A significant number of MS or MS/MS peaks were detected in the chromatograms of the supernatant of the RGM 2184 cultures; only four *m*/*z* peaks identified corresponded to the compounds previously described in EPF, and whose properties have been relatively well characterized. These compounds are oosporein, inflatin A, basiatin, and beauveriolide I and V. Their insecticidal (beauveriolide) and antimicrobial (oosporein) properties provide the basis for the mechanism of action employed by strain RGM 2184 to colonize the insect host and defend against opportunistic microorganisms.

Further studies on the purification and identification of the compounds secreted by RMG 2184, together with their evaluation in pest control, would contribute to the knowledge of new molecules for use in agriculture. The characterization of BGCs could offer the possibility of improving the efficacy of this strain by genetic modification to increase the production of these virulence factors. On the contrary, it could provide a new tool for direct control of insect pests by direct utilization of these factors, similar to a chemical pesticide. Both approaches offer the potential to increase the efficacy of entomopathogens or their products, thus aiding in the commercialization of microorganisms or their toxin as a biopesticide.

## Figures and Tables

**Table 1:** In silico ANIb, dDDH, and percentage of homologous genes encoding bioactive compounds values between *B. pseudobassiana* RGM 2184 and the other EPF strains.

Strain EPF	Order	Family	Subphylum	ANIb	DDH	Prob. DDH ≥70%	Homologues Genes Encoding for (%):			
							Exoenzymes	Toxins	NRPS	PKS
*Beauveria pseudobassiana* KACC 47484	*Hypocreales*	*Cordycipitaceae*	*Pezizomycotina*	96	70	78	98	80	100	95
*Beauveria brongniartii* RCEF 3172	*Hypocreales*	*Cordycipitaceae*	*Pezizomycotina*	89	37	1	95	70	80	90
*Beaubveria bassiana* ARSEF 2860	*Hypocreales*	*Cordycipitaceae*	*Pezizomycotina*	88	34	0	93	60	88	80
*Beauveria bassiana* ARSEF 1520	*Hypocreales*	*Cordycipitaceae*	*Pezizomycotina*	88	34	0	93	65	88	85
*Akanthomyces lecanii* RCEF 1005_R_	*Hypocreales*	*Cordycipitaceae*	*Pezizomycotina*	78	21	0	65	30	60	45
*Cordyceps javanica* IJ1G	*Hypocreales*	*Cordycipitaceae*	*Pezizomycotina*	78	21	0	61	35	68	45
*Cordyceps javanica* IJ2G_R_	*Hypocreales*	*Cordycipitaceae*	*Pezizomycotina*	78	21	0	61	35	68	45
*Cordyceps militaris* CM01_R_	*Hypocreales*	*Cordycipitaceae*	*Pezizomycotina*	78	21	0	54	25	72	70
*Akanthomyces lecanii* UM487	*Hypocreales*	*Cordycipitaceae*	*Pezizomycotina*	78	22	0	38	25	56	40
*Cordyceps fumosorosea* ARSEF 2679_R_	*Hypocreales*	*Cordycipitaceae*	*Pezizomycotina*	78	21	0	53	20	68	60
*Lecanicillium psalliotae* HWLR35	*Hypocreales*	*Cordycipitaceae*	*Pezizomycotina*	77	21	0	42	25	64	50
*Tolypocladium paradoxum* NRBC 100945_R_	*Hypocreales*	*Ophiocordycipitaceae*	*Pezizomycotina*	71	20	0	0	15	24	40
*Purpureocillium lilacinum* PLFJ-1_R_	*Hypocreales*	*Ophiocordycipitaceae*	*Pezizomycotina*	71	20	0	0	10	20	50
*Sporothrix insectorum* RCEF 264^R^	*Ophiostomatales*	*Ophiostomataceae*	*Pezizomycotina*	70	19	0	0	0	8	50
*Metarhizium album* ARSEF 1941_R_	*Hypocreales*	*Clavicipitaceae*	*Pezizomycotina*	70	20	0	0	10	24	45
*Moelleriella libera* RCEF 2490R	*Hypocreales*	*Clavicipitaceae*	*Pezizomycotina*	70	20	0	0	10	24	50
*Metarhizium rileyi* RCEF 4871	*Hypocreales*	*Clavicipitaceae*	*Pezizomycotina*	70	22	0	0	24	20	45
*Metarhizium rileyi* Cep018-CH2R	*Hypocreales*	*Clavicipitaceae*	*Pezizomycotina*	70	22	0	0	15	20	45
*Metarhizium brunneum* ARSEF 3297_R_	*Hypocreales*	*Clavicipitaceae*	*Pezizomycotina*	70	21	0	1	30	20	50
*Metarhizium robertsii* ARSEF 2575	*Hypocreales*	*Clavicipitaceae*	*Pezizomycotina*	70	22	0	0	20	20	50
*Metarhizium robertsii* ARSEF 23_R_	*Hypocreales*	*Clavicipitaceae*	*Pezizomycotina*	70	22	0	0	20	20	50
*Metarhizium anisopliae* ARSEF 549	*Hypocreales*	*Clavicipitaceae*	*Pezizomycotina*	70	21	0	1	20	20	50
*Metarhizium anisopliae* BRIP 53293	*Hypocreales*	*Clavicipitaceae*	*Pezizomycotina*	70	21	0	1	15	20	50
*Ophiocordyceps polyrhachis-furcata* BCC 54312_R_	*Hypocreales*	*Ophiocordycipitaceae*	*Pezizomycotina*	70	19	0	14	10	16	50
*Metarhizium acridum* CQMa 102_R_	*Hypocreales*	*Clavicipitaceae*	*Pezizomycotina*	70	21	0	2	20	20	45
*Aspergillus flavus* NRRL 118543	*Eurotiales*	*Aspergillaceae*	*Pezizomycotina*	67	20	0	0	0	16	60
*Aspergillus flavus* NRRL3357_R_	*Eurotiales*	*Aspergillaceae*	*Pezizomycotina*	67	19	0	0	0	16	55

R, representative genome published in NCBI; KACC, Korean Agricultural Culture Collection; ARCEF, Agricultural Research Service Collection of Entomopathogenic Fungal Cultures.

## Data Availability

This Whole Genome Shotgun project has been deposited to DDBJ/ENA/GenBank under accession JAKJXD000000000.

## Supplementary Materials

The following supporting information can be downloaded at: https://www.mdpi.com/article/10.3390/jof8030253/s1. Figure S1: Peak chromatogram detected from the metabolite extract obtained from the supernatant of the strain RGM 2184 culture medium; Table S1: List of COG used in the multilocus phylogenetic analysis; Table S2: Genome features of EPF strains; Table S3: List of extracellular enzymes predicted in the genome of strain RGM 2184; Table S4: List of NRPS and PKS predicted in the genome of strain RGM 2184; Table S5: Known BGR cluster predicted in strain RGM 2184; Table S6: Toxins encoded in the genome of strain RGM 2184; Table S7: Chromatographic peak obtained from MS analysis of the supernatant of the culture of strain RGM 2184 in M2; Table S8: Chromatographic peak obtained from MS analysis of the supernatant of the culture of strain RGM 2184 in YSM; Table S9: Chromatographic peak obtained from MS/MS analysis of the supernatant of the culture of strain RGM 2184 in YSM. Refs. [66,67,68,69,70,71,72,73,74,75,76,77,78,79,80,81] are cited in Table S2.
