# Supplementary material for: Genomic and Experimental Analysis of the Insecticidal Factors Secreted by the Entomopathogenic Fungus Beauveria pseudobassiana RGM 2184
Source: J Fungi (Basel). 2022 Mar 1;8(3):253. doi: 10.3390/jof8030253 (PMC8952764; doi:10.3390/jof8030253)

(a)

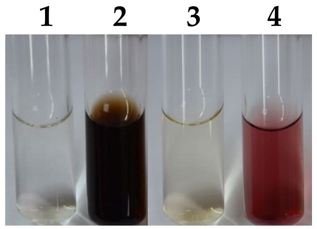

1: M2 medium
2: Supernatant extraction from RGM 2184 culture in
3: YSM growth medium
4: supernatant extraction from RGM 2184 culture in

(b)
(c)
(d)

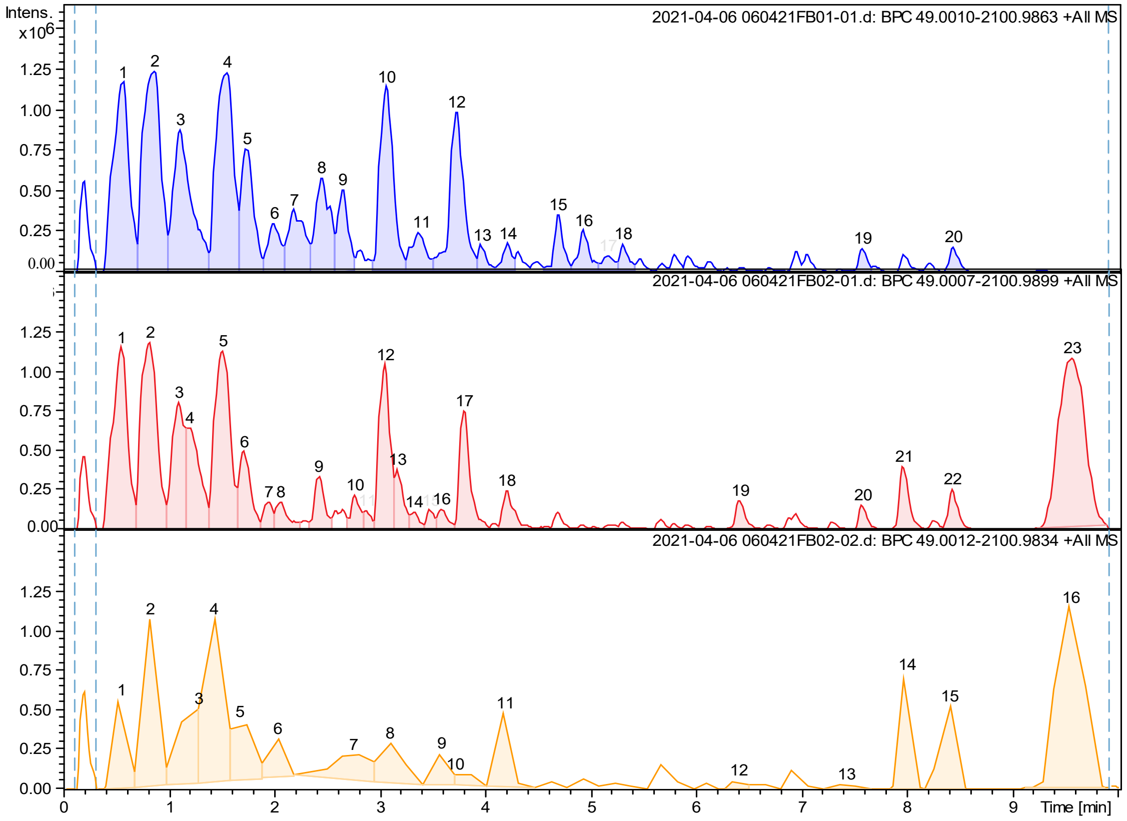

Supplement: Supplementary file 1 [file jof-08-00253-s001.zip › jof-1608862-supplementary/Figure S1.pdf]
