# Supplementary material for: Genomic and Experimental Analysis of the Insecticidal Factors Secreted by the Entomopathogenic Fungus Beauveria pseudobassiana RGM 2184
Source: J Fungi (Basel). 2022 Mar 1;8(3):253. doi: 10.3390/jof8030253 (PMC8952764; doi:10.3390/jof8030253)
Supplement: Supplementary file 1 [file jof-08-00253-s001.zip › jof-1608862-supplementary/Table S1.pdf]

Table S1. List of COG used in the multilocus phylogenetic analysis.

COG annotation	
COG00048	Ribosomal protein S12
COG00049	Ribosomal protein S7
COG00051	Ribosomal protein S10
COG00052	Ribosomal protein S2
COG00081	Ribosomal protein L1
COG00087	Ribosomal protein L3
COG00088	Ribosomal protein L4
COG00090	Ribosomal protein L2
COG00091	Ribosomal protein L22
COG00093	Ribosomal protein L14
COG00094	Ribosomal protein L5
COG00096	Ribosomal protein S8
COG00097	Ribosomal protein L6P/L9E
COG00098	Ribosomal protein S5
COG00099	Ribosomal protein S13
COG00100	Ribosomal protein S11
COG00102	Ribosomal protein L13
COG00103	Ribosomal protein S9
COG00184	Ribosomal protein S15P/S13E
COG00244	Ribosomal protein L10
COG00255	Ribosomal protein L29
COG00256	Ribosomal protein L18
COG00522	Ribosomal protein S4 and related proteins
COG01358	Ribosomal protein HS6-type (S12/L30/L7a)
