# Supplementary material for: Genomic and Experimental Analysis of the Insecticidal Factors Secreted by the Entomopathogenic Fungus Beauveria pseudobassiana RGM 2184
Source: J Fungi (Basel). 2022 Mar 1;8(3):253. doi: 10.3390/jof8030253 (PMC8952764; doi:10.3390/jof8030253)
Supplement: Supplementary file 1 [file jof-08-00253-s001.zip › jof-1608862-supplementary/Table S2.pdf]

Table S2. Genome features of EPF strains.

EPF strains	Size (Mb)	Coverage (fold)	N° scaffold (>1kb)	Scaffold N50 (Mb)	% G+C content	Protein-coding genes	Accession number Genebank	Ref.
B. pseudobassiana RGM 2184	34.5	60x	47	3.86	51.7	8469	JAKJXD00000000.1	
B. brongniartii RCEF 3172	32.5	73.46x	241	0.77	51.5	9595	AZHA00000000.1	[66]
B. pseudobassiana KACC 47484	34.5	200x	524	1.59	49.4	NA	LUDX00000000.1	[67]*
B. bassiana ARSEF 2860	33.7	28x	237	0.73	51.5	10.364	ADAH00000000.1	[53]
B. bassiana ARSEF 1520	40.0	50x	251	1.56	49.6	NA	JTCW00000000.1	[53]
A. flavus NRRL3357	37.0	70x	8	4.7	48.03	12009	CP059866.1-CP059873.1	[68]
A. nidulans NRRL 118543	37.2	100x	295	0.81	48	12818	NWUH00000000.2	[69]
A. lecanii RCEF 1005	35.6	81.94x	130	3.95	53.1	11030	AZHF00000000.1	[66]
A. lecanii UM487	32.6	87x	4750	0.021	51	8126	LUKN00000000.1	[70]
M. rileyi Cep018-CH2	31.8	341.9x	249	0.81	50,0	8944	SBHS00000000.1	[71]
M. rileyi RCEF 4871	32.0	107.34x	389	0.89	49.3	8764	AZHC00000000.1	[66]
M. brunneum ARSEF 3297	37.0	80.13x	92	1.82	51.5	10.689	AZNG00000000.1	[72]
M. album ARSEF 1941	30.4	117.07x	257	1.09	52.8	8472	AZHE00000000.1	[72]
M. acridum CQMa 102	38.1	107x	241	0.33	50	9.849	ADNI00000000.1	[73]
M. anisopliae BRIP 53293	38.7	357x	577	1.24	51.4	NA	APNB00000000.1	[74]
M. anisopliae ARSEF 549	38.5	98.34x	74	2.05	50.9	10.891	AZNF00000000.1	[72]
M. robertsii ARSEF 2575	40.3	25x	367	0.57	50.8	12384	JELW00000000.1	[75]
M. roberstsii ARSEF 23	39.0	147.33x	90	4.49	51.4	11688	ADNJ00000000.2	[73]
C. fumosorosea ARSEF 2679	33.0	86.99x	430	0.87	53.6	10.061	AZHB00000000.1	[66]
C. javanica IJ2G	34.97	199x	173	1.8	52.5	11.142	SPUL00000000.1	[76]
C. javanica IJ1G	34.9	338x	102	1.8	53.1	11.441	SPUK00000000.1	[76]
C. militaris CM01	32.3	100X	32	4.55	51.4	9.864	AEVU00000000.1	[77]
L. psalliotae HWLR35	36.1	80x	194	2.33	52.7	NA	PHFE00000000.1	[78]
S. insectorum RCEF 264	34.7	7037.0x	78	1.74	53.7	9.496	AZHD00000000.1	[66]
P. lilacinum PLFJ-1	38.5	152x	163	3.2	58.3	11763	LSBI00000000.1	[79]

O. polyrhachis-furcata BCC 54312	43.3	37x	68	2.98	43.3	10146	LKCN00000000.2	[80]
T. paradoxum NRBC 100945	27.6	80x	1138	0.08	57.9	8.983	PKSG00000000.1	[81]
M. libera RCEF 2490	30.9	76x	97	1.28	53.6	8461	AZGY00000000.1	[66]

*Unpublished
