# Supplementary material for: Genomic and Experimental Analysis of the Insecticidal Factors Secreted by the Entomopathogenic Fungus Beauveria pseudobassiana RGM 2184
Source: J Fungi (Basel). 2022 Mar 1;8(3):253. doi: 10.3390/jof8030253 (PMC8952764; doi:10.3390/jof8030253)
Supplement: Supplementary file 1 [file jof-08-00253-s001.zip › jof-1608862-supplementary/Table S3.pdf]

Table S3. List of extracellular enzymes predicted in the genome of strain RGM 2184.

Extracellular enzymes	Type of enzymes	Location			
		Scaffold	From	-	to
Alkaline serine protease ver112	Protease	1	3847462	-	3847963
Alkaline protease 1		1	4241508	-	4242988
Tripeptidyl-peptidase sed3		1	5590842	-	5591105
Glutamate carboxypeptidase 2		1	1479765	-	1482809
Alkaline protease		1	1714910	-	1716167
Extracellular metalloprotease		1	2499475	-	2500356
Neutral protease		2	550064	-	551175
Tripeptidyl-peptidase		2	791064	-	792129
Extracellular metalloprotease		2	1539371	-	1539610
Probable tripeptidyl-peptidase		2	1545350	-	1547192
Probable serine protease		2	353733	-	355446
Putative serine protease		2	2673672	-	2675392
peptidase complex catalytic		2	2932267	-	2932851
Subtilisin-like protease		2	3150662	-	3151084
Dipeptidyl peptidase		2	3164382	-	3169037
Tripeptidyl-peptidase		2	3223233	-	3224040
Metallocarboxypeptidase A-like protein		2	3496907	-	3498368
Tripeptidyl-peptidase sed2		3	4442095	-	4444211
Putative dipeptidase		3	1121498	-	1123044
Minor extracellular protease vpr		3	161134	-	164083
Tripeptidyl-peptidase		3	3726814	-	3728539
Cuticle-degrading protease		4	1173612	-	1174926
Probable aspartic-type endopeptidase		4	1654670	-	1656103
Subtilisin-like proteinase Spm1		4	1973365	-	1975001
Carboxypeptidase		4	2698558	-	2699316
Putative serine protease		5	532844	-	534008
Carboxypeptidase		5	974738	-	976358
Tripeptidyl-peptidase		5	2118967	-	2121809
Alkaline proteinase		5	2620367	-	2621332
Disintegrin and metalloproteinase		5	2311775	-	2314317
Tripeptidyl-peptidase SED1		6	1039600	-	1041346
Extracellular metalloprotease		6	1167071	-	1168139
Carboxypeptidase		6	1896663	-	1896950
Extracellular metalloprotease		6	2773076	-	2774099
Disintegrin and metalloproteinase		6	3056939	-	3058605
Carboxypeptidase		6	3306644	-	3308060
Peptidoglycan endopeptidase RipB		6	3583501	-	3583909
Leucine aminopeptidase		7	630309	-	631736
Carboxypeptidase		7	2402874	-	2404804

Intermediate cleaving peptidase		8	766734	-	767256
Tripeptidyl-peptidase		8	922567	-	924562
Carboxypeptidase		8	89572	-	90922
Protease		8	1149722	-	1152270
Minor extracellular protease vpr		11	253959	-	256735
Minor extracellular protease vpr		14	123525	-	126567
Carboxypeptidase B		14	182965	-	184813
Lipase 9	Lipase	1	4693577	-	4695016
Lipase 1		1	3248701	-	3250054
Lipase YOR059C		2	2350669	-	2352135
Lipase 3		2	71439	-	72293
Carboxylesterase		3	4056146	-	4057795
Lipase 2		5	904189	-	905904
Lipase atg15		5	243713	-	245654
Lipase		6	721579	-	722649
Carboxylesterase LipF		6	2920507	-	2921701
Lipase 5		6	3297909	-	3299370
Neutral ceramidase		7	1772262	-	1774975
Lipase 2		7	3092186	-	3093965
Neutral ceramidase		8	262869	-	267591
Glucan endo-1,3-beta-glucosidase eglC	Glycosidase	1	4997844	-	4999243
Endoglucanase EG-II		1	943544	-	944543
Glucan endo-1,3-beta-glucosidase A1		1	946057	-	947025
Xyloglucan-specific endo-beta-1,4-glucanase A		1	1668003	-	1669247
Probable beta-glucosidase M		2	4049120	-	4051798
Glucan 1,3-beta-glucosidase OS		3	4971359	-	4972027
Probable endo-1,3(4)-beta-glucanase		3	288950	-	291211
Probable endo-1,3(4)-beta-glucanase		3	2818532	-	2819613
Glucan endo-1,3-beta-glucosidase		3	3332295	-	3333211
Glucan endo-1,3-beta-glucosidase		3	3840814	-	3843132
Alpha/beta-glucosidase agdC		4	1550139	-	1552997
Alpha/beta-glucosidase agdC		4	2264971	-	2267682
Glucan endo-1,3-beta-glucosidase		4	268137	-	270295
Glucosidase 2 subunit alpha OS		4	2731472	-	2734401
Alpha/beta-glucosidase agdC		4	3043094	-	3046001
Beta-glucosidase btgE		4	3083111	-	3084977
Glucan endo-1,3-beta-glucosidase eglC		5	464887	-	468346
Beta-glucosidase F		6	715323	-	718341
Beta-galactosidase A		6	2262408	-	2263280
Glucan 1,3-beta-glucosidase		6	3118128	-	3121857
Glucan endo-1,6-beta-glucosidase B		6	3329068	-	3330420
Glycosidase Rv0584		6	3334812	-	3337101

Endo-beta-1,4-glucanase D		7	2105170	-	2106276
Glucan endo-1,3-alpha-glucosidase		8	480476	-	481160
Glucan endo-1,3-beta-glucosidase		8	324990	-	328136
Glucosidase		16	26552	-	28331
Glycosidase Rv0584		1	5135947	-	5138488
Glycosidase Rv0584		3	3919243	-	3921939
Glycosidase Rv0584		8	787460	-	791275
Acetylxylan esterase		1	1475945	-	1476786
O-GlcNAcase NagJ	Chitinase	3	4755715	-	4757014
Endo-beta-N-acetylglucosaminidase		4	611181	-	611813
Sporulation-specific chitinase		6	2068993	-	2070225
Lysophospholipase	Phospholipase	2	313790	-	317947
Hemolytic phospholipase C		2	3180919	-	3181856
Hemolytic phospholipase C		2	3258003	-	3258257
Lysophospholipase		3	3420754	-	3421606
Laccase-4	Laccase	2	3214792	-	3215318
Laccase-1		4	2452900	-	2454862
Oxidoreductase YgbJ	Other	3	3736576	-	3739952
Oxidoreductase DltE		8	67728	-	68629
UDP-2-acetamido-2-deoxy-3-oxo-D-glucuronate aminotransferase		1	5358141	-	5359706
Fumarate reductase		1	5421549	-	5422092
Oxoacyl-CoA reductase		1	3689511	-	3690464
Acetylxylan esterase O		2	3401483	-	3402499
Hydrolase		3	3775603	-	3777506
AB hydrolase superfamily protein C4A8.06c		4	752251	-	753426
Hydrolase Mb2248c		4	1019513	-	1021063
Hydrolase Mb2248c		4	2103670	-	2105316
epoxide hydrolase		5	432651	-	433628
Trehalase		5	2096854	-	2099008
Aromatic compound monooxygenase		5	2781924	-	2783343
Sorbitol dehydrogenase		6	1639525	-	1640204
aspartate-semialdehyde dehydrogenase		6	2199934	-	2201326
Prolyl 4-hydroxylase subunit alpha-2		6	3837654	-	3838503
