# Supplementary material for: Genomic and Experimental Analysis of the Insecticidal Factors Secreted by the Entomopathogenic Fungus Beauveria pseudobassiana RGM 2184
Source: J Fungi (Basel). 2022 Mar 1;8(3):253. doi: 10.3390/jof8030253 (PMC8952764; doi:10.3390/jof8030253)
Supplement: Supplementary file 1 [file jof-08-00253-s001.zip › jof-1608862-supplementary/Table S4.pdf]

Table S4. List of NRPS and PKS predicted in the genome of strain RGM 2184.

Enzyme	Location			
	Conting	From	-	to
Nonribosomal peptide synthetase	1	3916622	-	3920076
Nonribosomal peptide synthetase	1	3933879	-	3940625
Nonribosomal peptide synthetase	1	3940682	-	3943816
Polyketide synthase	1	6056861	-	6057482
Phenolphthiocerol synthesis polyketide synthase type I	2	4278566	-	4285005
Phthiocerol synthesis polyketide synthase type I	2	849139	-	855237
Phthiocerol synthesis polyketide synthase type I	2	849139	-	855237
Nonribosomal peptide synthetase	2	1582785	-	1583855
Conidial yellow pigment biosynthesis polyketide synthase	2	2074744	-	2077615
Conidial yellow pigment biosynthesis polyketide synthase	2	2078806	-	2080026
Nonribosomal peptide synthetase	2	3023261	-	3035891
Nonribosomal peptide synthetase	2	3498578	-	3502754
Nonribosomal peptide synthetase	2	453195	-	456640
Polyketide synthase	3	3969177	-	3970217
Phthiocerol synthesis polyketide synthase type I	3	2748268	-	2749366
Nonribosomal peptide synthetase	3	2918344	-	2920895
Lovastatin nonaketide synthase	3	2923546	-	2925625
Nonribosomal peptide synthetase	3	2929177	-	2939572
Granaticin polyketide synthase	3	3336223	-	3337135
Phthiocerol synthesis polyketide synthase type I	4	585226	-	592833
Nonribosomal peptide synthetase	4	2094871	-	2095420
Nonribosomal peptide synthetase	4	2095798	-	2097240
Nonribosomal peptide synthetase	4	2097868	-	2098170
Phthiocerol synthesis polyketide synthase type I	4	19086	-	24583
Nonribosomal peptide synthetase	5	437923	-	439041
Lovastatin nonaketide synthase	5	439389	-	441763
Nonribosomal peptide synthetase	5	442193	-	444795
Lovastatin nonaketide synthase	5	1881589	-	1882023
Polyketide synthase	5	271531	-	272343
Conidial yellow pigment biosynthesis polyketide synthase	6	886073	-	887467
Conidial yellow pigment biosynthesis polyketide synthase	6	887627	-	889226
Conidial yellow pigment biosynthesis polyketide synthase	6	889599	-	890192
Nonribosomal peptide synthetase	6	1999401	-	2004756
Nonribosomal peptide synthetase	6	2004806	-	2005066
Nonribosomal peptide synthetase	6	2296117	-	2296737
Phenolphthiocerol synthesis polyketide synthase type I	6	2968372	-	2970200
Nonribosomal peptide synthetase	6	3535171	-	3535943
Nonribosomal peptide synthetase	6	3536879	-	3537117
Conidial yellow pigment biosynthesis polyketide synthase	7	2154205	-	2160113

Phthiocerol synthesis polyketide synthase type I	7	2708878	-	2713579
Nonribosomal peptide synthetase	8	1567561	-	1571440
Nonribosomal peptide synthetase	8	1573780	-	1575568
Nonribosomal peptide synthetase	13	231551	-	231937
Nonribosomal peptide synthetase	13	234801	-	236528
