# Supplementary material for: Genomic and Experimental Analysis of the Insecticidal Factors Secreted by the Entomopathogenic Fungus Beauveria pseudobassiana RGM 2184
Source: J Fungi (Basel). 2022 Mar 1;8(3):253. doi: 10.3390/jof8030253 (PMC8952764; doi:10.3390/jof8030253)
Supplement: Supplementary file 1 [file jof-08-00253-s001.zip › jof-1608862-supplementary/Table S5.pdf]

Table S5. Known BGR cluster predicted in strain RGM 2184.

Metabolite	Gen	Localization				Gen length	Product	Identity/coverage (%)*
		Conting	From		to			
Oosporein	*opS10*	*7*	*2138290*	-	2141113	2.824 bp	Putative Heat-labile enterotoxin IIB, A chain	93/98
	opS11	*7*	*2143116*	-	2144767	1.652 bp	D-lactonohydrolase	93/98
	opS12	*7*	2145358	-	2146593	1.236 bp	Mitochondrial CorA family metal ion transporter	92/100
	opS13	*7*	2148519	-	2146739	1.780 bp	Cation transporter	89/100
	opS14	*7*	2149264	-	2153362	4.499 bp	Hydantoinase B/oxoprolinase	94/100
	opS1	*7*	2161333	-	2154205	7.129 bp	Polyketide synthase	90/100
	opS2	*7*	2163537	-	2162436	1.102 bp	MFS multidrug resistance transporter	93/100
	opS3	*7*	2165847	-	2168031	2.185 bp	Fungal specific transcription factor	89/100
	opS4	*7*	2169938	-	218360	1.579 bp	FAD binding domain-containing protein	92/89
	opS5	*7*	2170886	-	2173002	2.117 bp	Laccase 2	91/100
	opS6	*7*	2174103	-	2173368	736 bp	Glutation-S-transferase	90/100
	opS7	*7*	2174656	-	2175711	1.056 bp	Enzyme involved in oosporein biosynthesis.	88/100
Beauvericin	*orf1*	*1*	1077828	-	1080529	2.701 bp	Hypothetical protein	93/99
	orf2	*1*	1081349	-	1083416	2.067 bp	Cyclophilin-type peptidyl-prolyl cis-trans isomerase	96/100
	orf3	*1*	1084023	-	1084755	732 bp	Integral membrane protein, putative	94/100
	orf4	*1*	1085146	-	1084849	-297	Glycolate oxidase L-lactate 2-monooxygenase	97/100
	kivr	*1*	1086833	-	1088364	1.531 bp	Ketoisovalerate reductase	88/91
	bbBeas	*1*	1090776	-	1100033	9.257 bp	BEAS beauvericin nonribosomal cyclodepsipeptide synthetase	90/100
	orf5	*1*	1104355	-	1105183	829 bp	Hypothetical protein	86/100

	orf6	*1*	1108718	-	1106968	1.750 bp	Calreticulin	95/100
	orf7	*1*	1109651	-	1111439	1.789 bp	Methylmalonate semialdehyde dehydrogenase	95/100
	orf8	*1*	1112769	-	1114579	1.810 bp	Hypothetical protein	91/99
Desmethylbassianin	*dmbA*	*2*	3041985	-	3040348	1.638 bp	Dmb	91/99
	dmbB	*2*	3038314	-	3039955	1.642 bp	DmbB	84/100
	dmbC	*2*	3036563	-	3037731	1.168 bp	DmbC	99/100
	dmbS	*2*	3035893	-	3023261	12.632 bp	DmbS, polyketide synthase-nonribosomal peptide synthase	97/99
Beauveriolide	*besA*	7	2722951	-	273806	15.113 bp)	NRPS	87/99
	besD	7	2720026	-	2722303	(2.277 bp)	Acyl-CoA-ligase	88/99
	besC	7	2718296	-	2719742	(1.447 bp)	Acyltransferease	88/100
	besB	7	2708864-	-	2716823	(7.959 bp)	PKS	92/100

*Identity percentaje between *B. spseudobassiana* RGM2184 v/s *B. bassiana* ARSF 2860 gene
