# Supplementary material for: Genomic and Experimental Analysis of the Insecticidal Factors Secreted by the Entomopathogenic Fungus Beauveria pseudobassiana RGM 2184
Source: J Fungi (Basel). 2022 Mar 1;8(3):253. doi: 10.3390/jof8030253 (PMC8952764; doi:10.3390/jof8030253)
Supplement: Supplementary file 1 [file jof-08-00253-s001.zip › jof-1608862-supplementary/Table S6.pdf]

Table S6. Toxins encoded in the genome of strain RGM 2184.

Toxins	Localization			
	Scaffold	From	-	to
Pesticidal crystal protein cry3Ba	1	5984334	-	5985824
Pesticidal crystal protein cry3Aa	3	5136359	-	5138413
Pesticidal crystal protein cry1Db	4	1557464	-	1558953
Pesticidal crystal protein cry3Ba	6	303557	-	305090
Heat-labile enterotoxin alpha chain	1	36671	-	37624
Heat-labile enterotoxin alpha chain	3	5317662	-	5318601
Heat-labile enterotoxin alpha chain	3	2198347	-	2201858
Heat-labile enterotoxin alpha chain	3	2664052	-	2664552
Heat-labile enterotoxin alpha chain	4	847533	-	850115
Heat-labile enterotoxin alpha chain	7	2138379	-	2141121
Heat-labile enterotoxin alpha chain	7	2297686	-	2298072
Heat-labile enterotoxin alpha chain	8	979509	-	980459
Killer toxin subunits alpha/beta	1	1311156	-	1314702
Killer toxin subunits alpha/beta	14	9435	-	9802
Killer toxin subunits alpha/beta	5	1045132	-	1049280
Killer toxin subunits alpha/beta	7	665634	-	671690
Killer toxin subunits alpha/beta	7	2290131	-	2294447
Clostridium neurotoxin	1	3275568	-	3277812
Insecticide toxin TcdB	6	605554	-	608737
Zeta toxin	3	4133201	-	4141793
Zeta toxin	3	3547528	-	3548328
Zeta toxin	4	2284720	-	2287438
