# Supplementary material for: Genomic and Experimental Analysis of the Insecticidal Factors Secreted by the Entomopathogenic Fungus Beauveria pseudobassiana RGM 2184
Source: J Fungi (Basel). 2022 Mar 1;8(3):253. doi: 10.3390/jof8030253 (PMC8952764; doi:10.3390/jof8030253)
Supplement: Supplementary file 1 [file jof-08-00253-s001.zip › jof-1608862-supplementary/Table S7.pdf]

Table S7. Chromatographic peak obtained from MS analysis of the supernatant of the culture of strain RGM 2184 in M2.

Peak	t_R (min)	Width (min)	m/z	Molecular formula	Compound
1	0.57	0.36-0.71	168.0298	$C_7H_5NO_4$	Dipicolinic acid
2	0.86	0.71-1.00	182.0460	$C_6H_9NO_4$	No candidate
3	1.12	1.00-1.39	211.1439	No determinated	No candidate
4	1.55	1.39-1.67	196.0613	$C_9H_9NO_4$	No candidate
5	1.75	1.67-1.91	260.1127	$C_{11}H_{17}NO_6$	Mycosporine-alanine
6	2.00	1.91-2.11	227.0908	$C_9H_{14}O_7$	Inflatin C
7	2.20	2.11-2.35	195.0648	$C_{10}H_{10}O_4$	No candidate
8	2.46	2.35-2.58	227.0910	$C_9H_{16}O_5$	Catathelasmol E
9	2.66	2.58-2.77	227.0911	$C_{11}H_{14}O_5$	No candidate
10	3.08	2.94-3.26	274.1296	No determinated	No candidate
11	3.38	3.26-3.51	209.0805	$C_{11}H_{12}O_4$	No candidate
12	3.74	3.51-3.93	241.1072	$C_{12}H_{16}O_5$	No candidate
13	3.97	3.93-4.10	322.2010	$C_{18}H_{27}NO_4$	No candidate
14	4.22	4.10-4.29	177.0543	$C_{10}H_8O_3$	No candidate
15	4.70	4.58-4.83	336.2165	$C_{19}H_{29}NO_4$	No candidate
16	4.94	4.83-5.08	440.3116	$C_{23}H_{41}N_3O_5$	Beauveriolide V/VI
17	5.18	5.08-5.27	323.2342	$C_{20}H_{31}O_2$	No candidate
18	5.32	5.27-5.44	488.3118	$C_{22}H_{41}N_5O_7$	Beauverolide H/I/III
19	7.59	7.51-7.68	312.3257	No determinated	No candidate
20	8.46	8.35-8.74	427.3778	$C_{26}H_{50}O_4$	No candidate
