# Supplementary material for: Genomic and Experimental Analysis of the Insecticidal Factors Secreted by the Entomopathogenic Fungus Beauveria pseudobassiana RGM 2184
Source: J Fungi (Basel). 2022 Mar 1;8(3):253. doi: 10.3390/jof8030253 (PMC8952764; doi:10.3390/jof8030253)
Supplement: Supplementary file 1 [file jof-08-00253-s001.zip › jof-1608862-supplementary/Table S8.pdf]

Table S8. Chromatographic peak obtained from MS analysis of the supernatant of the culture of strain RGM 2184 in YSM.

Peak	t_R (min)	with (min)	m/z	Molecular formula	Compound
1	0.56	0.34-0.69	168.0299	$C_7H_5NO_4$	Dipicolinic acid
2	0.82	0.69-0.98	182.0457	$C_6H_9NO_4$	No candidate
3	1.10	0.98-1.17	211.1442	No determinated	No candidate
4	1.20	1.17-1.39	211.1441	$C_{11}H_{18}N_2O_2$	No candidate
5	1.52	1.39-1.66	196.0609	$C_9H_9NO_4$	No candidate
6	1.72	1.66-1.88	260.1129	$C_{11}H_{17}NO_6$	Mycosporine-alanine
7	1.95	1.88-2.01	291.1339	$C_{15}H_{18}N_2O_4$	No candidate
8	2.06	2.01-2.25	188.1280	No determinated	No candidate
9	2.43	2.33-2.55	222.1124	$C_{10}H_{17}NO_3$	No candidate
10	2.77	2.70-2.85	213.1120	$C_{11}H_{16}O_4$	No candidate
11	2.87	2.85-2.96	213.1120	$C_{11}H_{16}O_4$	No candidate
12	3.06	2.96-3.14	274.1293	No determinated	No candidate
13	3.17	3.14-3.29	301.1071	$C_{15}H_{18}O_5$	No candidate
14	3.33	3.29-3.43	331.1176	$C_{16}H_{20}O_6$	No candidate
15	3.49	3.43-3.55	250.1436	$C_{12}H_{21}NO_3$	No candidate
16	3.60	3.55-3.71	289.1073	$C_{14}H_{18}O_5$	No candidate
17	3.81	3.71-4.05	262.1440	$C_{15}H_{19}NO_3$	Bassiatin
18	4.21	4.14-4.49	177.0545	$C_{10}H_8O_3$	No candidate
19	6.42	6.26-6.63	331.2844	$C_{16}H_{20}O_6$	No candidate
20	7.58	7.46-7.82	391.2844	$C_{22}H_{40}O_4$	No candidate
21	7.98	7.82-8.19	279.1594	$C_{14}H_{24}O_4$	No candidate
22	8.44	8.36-8.74	427.3786	$C_{26}H_{50}O_4$	No candidate
23	9.57	9.27-9.91	547.4002	$C_{33}H_{54}O_6$	No candidate
