# Supplementary material for: Genomic and Experimental Analysis of the Insecticidal Factors Secreted by the Entomopathogenic Fungus Beauveria pseudobassiana RGM 2184
Source: J Fungi (Basel). 2022 Mar 1;8(3):253. doi: 10.3390/jof8030253 (PMC8952764; doi:10.3390/jof8030253)
Supplement: Supplementary file 1 [file jof-08-00253-s001.zip › jof-1608862-supplementary/Table S9.pdf]

Table S9. Chromatographic peak obtained from MS/MS analysis of the supernatant of the culture of strain RGM 2184 in YSM.

Peak (MS)	Peak (MS/MS)	t_R (min)	width (min)	m/z	Molecular formula	Compounds
1	6	0.54	0.53-0.55	168.0291	$C_7H_5NO_4$	Dipicolinic acid
2	21	0.84	0.83-0.85	182.0456	$C_8H_7NO_4$	4-methyl-2.6-pyridinedicarboxylic acid
2	22	0.85	0.83-0.87	174.1125	No determinated	No candidate
2	23	0.86	0.83-0.88	307.0451	$C_{14}H_{10}O_8$	Oosporein
2	24	0.87	0.83-0.90	234.0396	$C_{11}H_7NO_5$	8-hydroxyquinoline-2.4-dicarboxylic acid
2	26	0.88	0.83-0.93	243.1338	$C_9H_{20}N_2O_4$	No candidate
2	27	0.89	0.83-0.95	253.0706	No determinated	No candidate
3	37	1.15	1.14-1.15	211.1439	No determinated	No candidate
3	38	1.15	1.14-1.17	208.0961	$C_{11}H_{13}NO_3$	N-Acetyl-DL-phenylalanine
4	45	1.30	1.29-1.31	169.0496	$C_8H_8O_4$	Orsellinic acid
5	61	1.60	1.59-1.61	196.0603	$C_9H_9NO_4$	Dimethyl-2.6-pyridinedicarboxylic acid
5	62	1.61	1.59-1.63	243.0433	$C_9H_{10}N_2O_4S$	No candidate
5	63	1.62	1.59-1.64	257.1496	No determinated	No candidate
5	65	1.64	1.59-1.68	248.0551	$C_{12}H_9NO_5$	No candidate
5	66	1.64	1.59-1.69	238.1070	No determinated	No candidate
5	69	1.75	1.74-1.76	260.1130	$C_{11}H_{17}NO_6$	Mycosporine-alanine
6	77	1.91	1.90-1.91	291.1340	$C_{13}H_{20}N_2O_4$	No candidate
6	79	1.92	1.90-1.95	223.1074	$C_{11}H_{14}N_2O_3$	Phenylalanylglycine
6	80	1.93	1.90-1.96	320.1338	$C_{14}H_{17}N_5O_4$	No candidate
6	81	1.94	1.90-1.98	218.1382	No determinated	No candidate
6	101	2.36	2.35-2.37	340.1028	$C_{15}H_{17}NO_8$	2.3-Dihydroxy-quinoline-4-O-beta-D-glucopyranoside
6	102	2.37	2.35-2.39	304.1391	$C_{14}H_{21}N_2O_4$	No candidate
7	103	2.38	2.35-2.40	222.1121	$C_{12}H_{15}NO_3$	No candidate
7	125	2.82	2.81-2.83	260.1492	No determinated	No candidate
7	126	2.83	2.81-2.84	213.1118	$C_9H_{18}O_4$	No candidate

7	128	2.84	2.81-2.88	430.1494	No determinated	No candidate
7	129	2.85	2.81-2.89	388.1032	No determinated	No candidate
7	131	2.87	2.81-2.93	302.0659	$C_{15}H_{11}NO_6$	Bassiatin / Lateritin
8	140	3.12	3.11-3.13	274.1283	$C_{11}H_{17}N_5O_2$	No candidate
8	141	3.13	3.11-3.15	301.1070	$C_{17}H_{16}O_5$	No candidate
8	144	3.16	3.11-3.20	294.1334	$C_{13}H_{21}NO_5$	No candidate
8	148	3.27	3.27-3.28	331.1179	$C_{18}H_{18}O_6$	No candidate
9	157	3.43	3.42-3.45	250.1431	No determinated	No candidate
9	159	3.45	3.42-3.49	448.1241	No determinated	No candidate
9	161	3.47	3.42-3.52	376.1400	No determinated	No candidate
9	164	3.58	3.57-3.59	289.1072	$C_{16}H_{16}O_5$	No candidate
9	165	3.59	3.57-3.60	275.1391	$C_{15}H_{18}N_2O_3$	No candidate
10	180	3.88	3.87-3.89	262.1436	$C_{15}H_{19}NO_3$	Bassiatin
11	196	4.19	4.18-4.20	177.0546	$C_{10}H_8O_3$	No candidate
12	306	6.38	6.36-6.41	348.3109	$C_{19}H_{41}NO_4$	No candidate
12	307	6.39	6.36-6.43	676.4992	$C_{35}H_{69}N_3O_7S$	No candidate
14	381	7.99	7.98-8.00	279.1593	$C_{16}H_{22}O_4$	No candidate
14	384	8.02	7.98-8.06	408.3106	$C_{24}H_{41}NO_4$	No candidate
15	399	8.43	8.42-8.44	427.3784	$C_{26}H_{50}O_4$	No candidate
15	400	8.44	8.42-8.45	444.4050	$C_{26}H_{53}NO_4$	No candidate

LC, liquid chromatography.
